# Serum cardiac markers are inversely associated with VO_2_max of amateur athletes in response to endurance training adaptations

**DOI:** 10.1136/bmjsem-2019-000537

**Published:** 2019-04-09

**Authors:** Gashaw Tesema, Mala George, Soumitra Mondal, D Mathivana

**Affiliations:** 1 Department of Sport Science, College of Natural and Computational Science, Mizan-Tepi University, Mizan, Ethiopia; 2 Department of Sport Science, College of Natural and Computetional Science, Mekelle University, Mekelle, Ethiopia; 3 Department of Biochemistry, College of Health Sciences, Mekelle University, Mekelle, Ethiopia; 4 Department of Sport Science, College of Natural and Computational Science, Mekelle University, Mekelle, Ethiopia

**Keywords:** amateur athletes, cardiac muscle damage, endurance training, serum markers, VO2max

## Abstract

**Background:**

The influence of endurance training intensity and adaptation on serum cardiac markers is poorly understood and controversial; however, no enough data observed the association of serum cardiac markers with VO_2_max. Therefore, we aimed to investigate whether serum cardiac markers are associated with maximum oxygen consumption (VO_2_max) in response to 12-week endurance training on amateur athletes.

**Methods:**

15 apparently healthy male amateur athletes with 19.47 ± 1.30 years of age were recruited and participated in endurance training with 70%–80% maximal heart rate intensity for 35 min per session for the first week and 2 min increments each week from the second to the last week for a period of 12 weeks. VO_2_max and serum cardiac markers (lactate dehydrogenase [LDH], creatine kinase myocardial band (CK-MB) and cardiac troponin I [CTnI]) were assessed at the beginning of the training and after 12-week endurance training.

**Results:**

The result of CTnI indicated significantly (p < 0.01) and inversely (r = − 0.466) correlated with VO_2_max and CK-MB indicated significantly (p < 0.01) and inversely associated with VO_2_max (r = − 0.536) with moderate relationship. However, we did not find a significant association on LDH (p > 0.05) with VO_2_max in response to endurance training adaptation.

**Conclusion:**

Our finding confirms our hypothesis that serum cardiac markers are inversely associated with estimated VO_2_max in response to endurance training adaptation.

**What are the new findings?**

- Serum cardiac markers and maximum oxygen consumption have inverse relationship in young amateur moderately trained athletes.
- Gradual training intensity adaptation results in decreased serum cardiac markers.
- Increased serum cardiac markers are dependent on adaptation of training.

**How might it impact on clinical practice in the near future?**

- Elevation of serum cardiac markers in young, moderately trained amateur athletes may not be pathological rather it may be due to training experience, genetic factor and extreme intensity as well as training duration which needs further considerations.

## Introduction

Endurance training causes muscle damage, fatigue and muscle pain.^1 2^ This damage can be linked to large artery wall stiffening, cartilage and muscle damage,^3^ atrial fibrillation as well as left and right ventricular dysfunction^4^ and systemic inflammatory reaction^5^ resulting in elevated serum muscle damage markers. A study indicates seven continuous days of training did not increase incidences of muscle damage as compared with the group performed only one session of the training^6^ and this is attributed to adaptation of the stress caused by the training.^6^ Adaptations of training stress are best explained by maximum oxygen consumption (VO_2_max) as the single indicator of cardiorespiratory fitness. In line with this, researchers^7^ suggested the time course of the training related to muscle fibre damage, pain, as well as creatine kinase (CK), cardiac troponin I (CTnI) and lactate dehydrogenase (LDH) release. However, there are no evidences that indicate the association of serum cardiac markers and VO_2_max of amateur athletes in response to endurance training adaptation.

Moreover, studies reported serum cardiac markers (LDH, CK myocardial band [CK-MB] and CTnI) are not significantly correlated with VO_2_max,^8^ a measure of endurance training adaptation, which is a more accurate and reproducible predictor of cardiovascular outcomes.^9^ Conversely, a study reported muscle damage markers are inversely related to VO_2_max.^10^ Besides, the prevalence of elevated CTn is associated with inexperienced athletes than experienced athletes.^11^ This difference might strongly related to previous training experience^12^ and age^10^ of an athlete. Moreover, a 12-week soccer training with 80% (maximal heart rate, HRmax) average intensity: reported increased VO_2_max and inversely decreased CK-MB at the end of the 12-week training.^13^

However, there is no enough data observed the association of serum cardiac markers with VO_2_max in response to 12-week endurance training adaptation of amateur athletes. Moreover, there is a lack of prospective studies that observe the relationship of high intensity endurance training to exercise-induced cardiac remodelling.^14^ Thus, since the athletes are exposed for elevated serum cardiac markers as a result of endurance training, it helps them to understand the level of serum markers in response to adaptation and preventing overtraining for monitoring fitness and enhancing cardiac health.

The aim of this study was to examine the association between serum cardiac markers and VO_2_max in response to chronic endurance training on amateur athletes. Thus, we have applied high intensity endurance training at 70%–80% HRmax with duration of 35 min per session for the first week and 2 min increments each week from the second to the last week for a period of 12 weeks. Consequently, we hypothesised tht serum cardiac markers are inversely associated with estimated VO_2_max because of endurance training adaptation.

## Methods

### Study area

This study was conducted in Bahir Dar, located about 578 km north-northwest of Addis Ababa-Ethiopia. It has an altitude of 1840 m above the sea level and within latitude and longitude of 11°36′N 37°23′E coordinates. While the annual average temperature is 25°C–32°C, with an average humidity of 58%.^15^

### Study population

In all, 15 apparently healthy male amateur moderately trained athletes within 18–25 years of age were selected randomly from Bahir Dar University sport academy students. The sample size was based on similar studies done before^16^ using the following equations which considers 80% power and p≤0.05, where n=sample size needed, σ^∗2^ T is variance of the treatment, σ^∗2^ R is variance of the reference, δ is the maximum percent of no clinical importance, CVT is the coefficient of variations of the treatment and CVR is the coefficient of variations of the reference:

$$n=\frac{\left(z\alpha +z\beta /2{)}^{2}(\sigma_{T}^{*}2+\sigma_{R}^{*}2\right)}{(\delta -/CVT-CVR/{)}^{2}}|$$

Consequently, a validated physical activity readiness questionnaire^17^ was used to evaluate conditions that may prohibit participants from practising in endurance training. Therefore, participants that reported problems in their health (heart dysfunction and chest pain during exercise) and physical conditions (loss of balance, consciousness and bone or joint problem) and are currently taking anti-hypertensive drugs were excluded from the study. In addition, smokers and alcoholics were also excluded from participating in the study due to reported influences on both metabolic and physical performance biomarkers.^18 19^

### Study design

A single group pre–post design was used in this study. In all, 15 subjects selected and place to high intensity endurance training. VO_2_max and serum markers of muscle damage were assessed at the beginning of the training and after 12-week endurance training. High intensity endurance training was done at 70%–80% HRmax 35 min per session for the first week and 2 min increments each week from the second to the last week for a period of 12 weeks.

### Training protocol and ethical approval

This field-based study conducted based on the protocol specified here. One professional athletics coach assigned to the experiment. Five minutes of warming up and stretching exercise are given at the beginning of the training. The participants were performed at 70%–80% HRmax, a continuous endurance running in the open field track for 35 min per session for the first week and 2 min increments each week from the second to the last week. The training intensity was monitored with polar heart rate monitor throughout the entire session. The heart rate monitor adjusted to the designed percent maximum heart rate. Therefore, the beep sound of the heart rate monitor guided us if it is below and above the designed % HRmax to adjust the heart rate drift throughout the training. Five minutes of cooling dawn training were also given at the end of each training time for 3 days per week and it continued for 12 weeks aimed to observe chronic effects of the training on serum cardiac markers and VO_2_max. Participants were instructed not to participate in other exercise training and to continue the usual habit of nutrition throughout the experiment.

Ethical approval was obtained from Institutional Research Ethics Review Committee of Mekelle University conformed to the 1975 Declaration of Helsinki. Written consent was delivered to the participants and they were informed about the objectives of the study. Participation in this study was voluntary and their right not to participate was respected. Confidentiality and anonymity were also ensured.

### Assessment of VO_2_max

VO_2_max of subjects was estimated using 3 min step test. Subjects stepped up and down a 16.5 inch stepping bench with a rate of 96 steps per min for 3 min in time of metronome beep sound. Five seconds after exactly 3 min pulse was taken from radial artery with heart rate monitor tied around the wrist. Finally, maximal aerobic power (VO_2_max) was calculated using the equation VO_2_max=111.33 − (0.42×maximum pulse measured 5 s after stepping for 3 min)^20^ and reported in (mL/kg/min).

### Assays of serum cardiac markers

Blood samples of 5 mL were collected just before training (pretest) from each participant. Then, post-training samples collected 4 hours after the final training session at the end of 12-week endurance training. Since the peak cardiometabolic markers have been achieved 3–4 hours after training time.^21 22^ Blood was taken from an antecubital vein using vacutainer serum separator tube containing blood clotting accelerant gel. The serum was separated by centrifugation of the blood sample at 4000 rpm (revolution per minute) for 3 min and stored at −20°C until analysis. The level of LDH in U/L was measured using a spectrophotometric assay with BS-2E chemistry analyzer according to the guidelines of the International Federation of Clinical Chemistry (Beckman Coulter, Krefeld, Germany). The levels of cTnI band in ng/mL and CK-MB in ng/mL were measured by chemiluminescence immunoassay using Maglumi 800 fully automated chemiluminescence immunoassay analyzer.

### Data analysis

Analysis of the data obtained from the study inferential statistics was employed. To evaluate whether or not the data are normally distributed, the Kolmogorov-Smirnov and Shapiro-Wilk^23 24^ normality tests were applied. To identify the presence of any significant relationships between variables, Pearson's correlation analysis was used. IBM-SPSS V.20 packages were used to analyse the data. Variables were expressed as Pearson’s correlation coefficient (r) and probability value (p). In all cases, P<0.05 was accepted significant.

## Results

The Kolmogorov-Smirnov and Shapiro-Wilk’s normality tests^23 24^ indicate (p>0.05) and visual inspection of their histograms, normal Q–Q plots and box plots showed that the data were approximately normally distributed. General characteristics of participants in mean±SD were age in years (19.47±1.30), height in metres (1.67±0.07), body mass in kg (53.96±5.67) and body mass index in kg/m^2^ (19.31±1.41).

Serum cardiac markers correlation results with estimated VO_2_max in response to 12-week endurance training are shown in table 1. Value of CTnI with 12-week endurance training was significantly (p<0.01) and inversely correlated with estimated VO_2_max (r=−0.466) indicating low relationship. Moreover, CK-MB was statistically significant (p<0.01) and inversely associated with estimated VO_2_max (r=−0.536) with moderate relationship. Furthermore, CTnI and LDH showed significant (p<0.05) and positive association with CK-MB. However, we did not find a significant association of LDH with VO_2_max (r=−0.23) and CTnI in response to endurance training adaptation in amateur athletes.

**Table 1:** Serum cardiac muscle damage markers correlation result with VO_2_max

Pearson correlations		VO_2_max	CK-MB	CTnI	LDH
VO_2_max	Pearson’s r	–			
	P value	–			
CK-MB	Pearson’s r	−0.536**	–		
	P value	0.002	–		
CTnl	Pearson’s r	−0.466**	0.362*	–	
	P value	0.009	0.050	–	
LDH	Pearson’s r	−0.236	0.368*	0.066	–
	P value	0.209	0.045	0.728	–

*p<0.05, **p<0.01, ***p<0.001.

CK-MB, creatine kinase myocardial band;CTnI, Cardiac troponin I band;LDH, Lactate dehydrogenase;Vo_2_max, maximum oxygen consumption.

## Discussion

The purpose of this study was to examine the association between serum cardiac markers and VO_2_max in response to chronic endurance training on amateur athletes so as to consider cardiac health. The main findings of the study support our hypothesis that serum cardiac markers are inversely associated with estimated VO_2_max in response to endurance training adaptation. Consequently, serum cardiac markers (CTnI and CK-MB) in response to 12-week endurance training indicated a statistically significant (p<0.01) association with estimated VO_2_max. However, we did not find a significant association on LDH (p>0.05) with estimated VO_2_max in response to endurance training adaptation.

CTn and VO_2_max are important factors in the study of cardiac muscle damage markers and cardiorespiratory fitness. Consequently, our study confirmed a statistically significant and inverse associations of CTnI with estimated VO_2_max. Consistently, our study reported CTnI is inversely related to VO_2_max.^10^ The reason might be adaptation of the stress caused by chronic endurance training.^6^ Conversely, a study^8^ reported CTnI has no relation with VO_2_max. This disparity could be attributed to training experiences of the athletes.^2 11^

Recent evidences reported increased VO_2_max and inversely decreased CK-MB at the end of the 12-week training^13^ with 80% (HR max) average intensity. It supports our finding of CK-MB is significantly and inversely associated with estimated VO_2_max in response to endurance training adaptation. Similarly, a study reported an inverse relationship between cardiac markers and VO_2_max.^10^ On the contrary, CK-MB is not significantly correlated with VO_2_max.^8^ The variation of results between studies might be training experience of athletes^12^ and age.^10^

Changes in serum cardiac muscle damage markers can be influenced by gender, age, different training intensity, training method differences and in this study, we used calculated or estimated VO_2_max rather than measured VO_2_max which was the limitation of our study design. Thus, further study is needed regarding the fore mentioned points. Nevertheless, we can see training experience effect with gradual increment in training duration for 12 weeks improved VO_2_max and reduced serum cardiac markers. Therefore, these factors should be considered in designing training to improve cardiorespiratory fitness and maintain cardiovascular health.

## Conclusion

In conclusion, serum cardiac markers, CTnI band and CK-MB, are significantly and inversely associated with estimated VO_2_max in amateur athletes in response to 12-week endurance training. These findings add to the growing body of evidence linking between cardiac damage markers to cardiorespiratory fitness. Indicating gradual adaptation reduces serum cardiac marker concentration after exercise.
